# Supplementary material for: Floristic diversity and its relationships with human land use varied regionally during the Holocene
Source: Nat Ecol Evol. 2024 Jul 8;8(8):1459–71. doi: 10.1038/s41559-024-02457-x (PMC11310077; doi:10.1038/s41559-024-02457-x)
Supplement: Supplementary file 1 — Supplementary Tables 1–4 and Note 1. [file 41559_2024_2457_MOESM1_ESM.pdf]

nature ecology & evolution

Supplementary information

https://doi.org/10.1038/s41559-024-02457-x

# Floristic diversity and its relationships with human land use varied regionally during the Holocene

In the format provided by the authors and unedited

**Supplementary Table 1: Mean *p*-values for continental smooths across all 1000 resamples and metrics**. African and Australasian heterogeneity were not modelled and are therefore not included.

| **Smooth** | **Turnover (BC)** | **Richness** | **Evenness** | **Turnover (Jacc.)** |
|---|---|---|---|---|
| s(age_draw):continent Africa | 0.1347 | <0.001 | <0.001 | 0.003 |
| s(age_draw):continent Asia | 0.0017 | 0.0301 | <0.001 | 0.0416 |
| s(age_draw):continent Australasia | 0.0772 | <0.001 | <0.001 | 0.0278 |
| s(age_draw):continent Europe | <0.001 | <0.001 | <0.001 | <0.001 |
| s(age_draw):continent South_America | <0.001 | 0.002 | 0.0049 | 0.0119 |
| s(age_draw):continent North_America | <0.001 | <0.001 | <0.001 | <0.001 |

**SupplementaryTable 2: Summary of AIC differences between models fit with (`global_continent`) and without (`global`) continental smooths**. Median and ranges of AIC differences across all 1000 resamples for each metric. A significant improvement is judged by a reduction in AIC of greater than two. The vast majority of the 1,000 resamples across all metrics performed better with a continental smooth than without. E.g., for evenness, 96.1% of models performed significantly better with a separate smooth for continent included, compared to the same model with only a global smooth. African and Australasian heterogeneity were not modelled and are therefore not included.

| Metric | Model | Per cent "best" model | Median AIC difference | AIC diff. range |
|---|---|---|---|---|
| Evenness | global | 3.8 | -364 | -11 - -6391338286 |
| Evenness | global_continent | 96.1 | -2665 | -22 - -2231521085 |
| Richness | global | 0.1 | -828320381 | -828320381 - -828320381 |
| Richness | global_continent | 99.9 | -4675 | -270 - -14805 |
| Turnover (BC) | global | 0.2 | -5 | -5 - -6 |
| Turnover (BC) | global_continent | 99.8 | -466 | -29 - -982 |
| Turnover (Jacc.) | global_continent | 100 | -204 | -139 - -281 |

**SupplementaryTable 3: Summary of AIC differences between European and North American models fit with and without biome smooths**. Median and ranges of AIC differences across all 1000 resamples for each metric. A significant improvement is judged by a reduction in AIC of greater than two. The vast majority of the 1,000 resamples across all metrics and continents performed better with a biome smooth than without.

| Metric | Model | Per cent "best" model | Median AIC difference | AIC diff. range |
|---|---|---|---|---|
| Evenness | continent | 0.1 | -219 | -219 - -219 |
| Evenness | continent_biome | 99.9 | -2697 | -158 - -5884 |
| Richness | continent_biome | 100 | -477 | -357 - -624 |
| Turnover (BC) | continent_biome | 100 | -107 | -19 - -277 |
| Turnover (Jacc.) | continent_biome | 100 | -230 | -125 - -344 |
| Evenness | continent | 0.4 | -1699289 | -169 - -1069221310 |
| Evenness | continent_biome | 99.6 | -966 | -33 - -198908839860 |
| Richness | continent_biome | 100 | -280 | -220 - -348 |
| Turnover (BC) | continent | 0.1 | -11 | -11 - -11 |
| Turnover (BC) | continent_biome | 99.9 | -89 | -9 - -157 |
| Turnover (Jacc.) | continent | 0.1 | -6 | -6 - -6 |
| Turnover (Jacc.) | continent_biome | 99.8 | -39 | -2 - -91 |

**SupplementaryTable 4: Mean *p*-values for biome smooths in European and North American models across all 1000 resamples and metrics**.

| Region | Smooth | Turnover (BC) | Richness | Evenness | Turnover (Jacc.) |
|---|---|---|---|---|---|
| Europe | s(age_draw):BIOME Tundra | 0.0031 | <0.001 | <0.001 | 0.2862 |
| Europe | s(age_draw):BIOME Mediterranean Forests, Woodlands & Scrub | <0.001 | <0.001 | <0.001 | <0.001 |
| Europe | s(age_draw):BIOME Temperate Broadleaf & Mixed Forests | <0.001 | <0.001 | <0.001 | <0.001 |
| Europe | s(age_draw):BIOME Temperate Conifer Forests | <0.001 | <0.001 | <0.001 | <0.001 |
| Europe | s(age_draw):BIOME Boreal Forests/Taiga | 0.0081 | <0.001 | <0.001 | <0.001 |
| North_America | s(age_draw):BIOME Tundra | <0.001 | <0.001 | <0.001 | 0.0081 |
| North_America | s(age_draw):BIOME Temperate Broadleaf & Mixed Forests | <0.001 | <0.001 | <0.001 | <0.001 |
| North_America | s(age_draw):BIOME Temperate Conifer Forests | <0.001 | 0.7706 | <0.001 | <0.001 |
| North_America | s(age_draw):BIOME Boreal Forests/Taiga | <0.001 | <0.001 | <0.001 | 0.0063 |
| North_America | s(age_draw):BIOME Temperate Grasslands, Savannas & Shrublands | <0.001 | 0.0022 | <0.001 | 0.2491 |

**Supplementary Note 1. Pollen diversity methods description.**

**Compositional turnover rate**

Bray-Curtis

For pollen samples $U$ and $V$, Bray-Curtis dissimilarity ($BC_{UV}$) is calculated as,

$$BC_{UV} = \frac{\sum_{j=1}^{S} \left| x_{Uj} - x_{Vj} \right|}{\sum_{j=1}^{S} \left| x_{Uj} + x_{Vj} \right|}$$

where $x_{Uj}$ and $x_{Vj}$ are the abundance values of pollen type $j$ in plots $U$ and $V$, respectively, and $S$ is the total number of pollen types identified across the two samples.

Jaccard

For pollen samples $U$ and $V$, Jaccard dissimilarity ($Jacc_{UV}$) is calculated as,

$$Jacc_{UV} = 1 - \frac{| U \cap V |}{| U \cup V |}$$

where ∩ represents 'intersection' and ∪ represents 'union'.

Turnover interval-adjustment

We repeated the following procedure on both the Bray-Curtis and Jaccard turnover values. To calculate turnover adjusted for the time interval between two pollen samples, we computed i) the dissimilarity between all pairs of samples in each pollen record and ii) the associated time differences between them. We then regressed the turnover values against the logarithm of the time-difference values from all sites using a beta regression, `turnover ~ log(time_diff)`, while controlling for heteroskedasticity (the variance of turnover values increases as the time interval between samples increases) by modelling the dispersion using `time_diff`. We fit this model in **`glmmTMB`**[1]. We then subtracted the raw pairwise turnover value from that which is expected given the time interval between the two samples, resulting in a measure of turnover adjusted for time interval; our compositional turnover rate.

**Richness**

The rarefied richness of a pollen sample can potentially be influenced by a number of considerations (e.g., whether a pollen sample is representative of the wider pollen assemblage, that pollen samples are similar within and between pollen records) and there are a number of potential associated errors and uncertainties [e.g., different pollen analytical techniques, the interplay between the evenness and richness of pollen samples (see refs.[2–4] for full discussion)]. Removing every possible source of uncertainty is impossible[5], but our resampling procedure and our use of a standardised pollen dataset go some way to minimise these sources of variation. Some suggest that pollen-type rarefied richness estimates from within similar climate/vegetation zones (a criterion satisfied by our biome-scale analysis) represent plant richness reliably, but that non-similar places and times should be compared with caution[6]. Despite the potential challenges of interpreting pollen diversity data, this study builds on works that use rarefied richness to investigate Holocene plant diversity, at a range of spatial scales[5,7–10].

Pollen grains are rarely resolved to individual species. Coupled with this, some pollen types are harmonised to Family, some to Genus and few to Species. Our measure of richness therefore represents taxonomic and functional richness (members of the same genus/family commonly share certain functional attributes and support overlapping insect, herbivore and fungal communities), rather than species richness.

**Evenness**

Pielou's evenness[11] ($J$) is calculated as,

$$J = \frac{H'}{\log(S)}$$

where $S$ is the total number of pollen types in the sample (of 300 grains here), $\log$ represents a logarithm and $H'$ is the Shannon Index[12], which characterises how unpredictable an observation is. The Shannon Index is calculated as,

$$H' = -\sum_{i=1}^{S} p_i \log p_i$$

where $p_i$ is the proportion of pollen type $i$ in the pollen sample and $S$ is the number of pollen types in the sample. Throughout, we use the natural logarithm.

Pollen productivity and dispersal mechanisms vary between plants, which can confound attempts to understand, by inference, the diversities of plant communities the pollen spectra represent[3,13]. In samples with low numbers of identified pollen grains, high pollen producing plants are more likely to dominate the sample, which can lead to more uneven samples (i.e., samples dominated by few pollen types) and a greater chance that pollen richness is underestimated[14,15]. Pollen evenness and richness have been shown to be positively correlated in some studies, especially in samples with low numbers of identified pollen grains[13,15]. Despite this, other work has demonstrated that the two are not always linked[3].

**Heterogeneity**

Firstly, for each region (continent/biome), we filtered out all time bins with fewer than ten pollen records present. Then, to ensure that each pollen record was represented only once in each 500-year time bin, we randomly resampled a single pollen sample from each pollen record. To avoid sampling effort confounding the result (spatial beta diversity scales with the number of sites - here pollen records - included in an analysis) we then resampled from each time bin the minimum number of pollen records present in the least well represented time bin across all continents and biomes (necessarily above the minimum inclusion number of ten) and calculated the multi-site, abundance-based Bray-Curtis dissimilarity of these pollen records using the `beta.multi.abund` function and also the multi-site, incidence-based Jaccard dissimilarity using the `beta.multi` function, both from the **`betapart`** package[16]. We repeated this procedure (resample a single sample from each pollen record per bin, resample from this set the minimum number of records present across all bins, compute Jaccard and Bray-Curtis) 100 times for each time bin and calculated the mean compositional dissimilarity across the 100 replicates.

These heterogeneity analyses result in a mean Bray-Curtis and a mean Jaccard value per 500 year time bin, a maximum total of 23 values for each index per region. Each of these values is the mean of 100 replicates of the multi-site dissimilarity computed over all pollen records present in each time bin. We repeat this procedure 1,000 times to include the variation inherent to both the age-depth modelling and also the pollen resampling (downsampling each sample to 300 grains) procedures. For the other diversity metrics (turnover, richness, evenness), we used GAMs to estimate smoothed diversity trends through time (below). Due to the much smaller amount of data and the danger of overfitting, we instead present the distribution of values created by the 1,000 resamples in Figs. 2 & 3 and Extended Data Fig. 2.

Supplementary references.


1. Brooks, M. E. *et al.* glmmTMB Balances Speed and Flexibility Among Packages for Zero-inflated Generalized Linear Mixed Modeling. *R J.* **9**, 378–400 (2017).
2. Birks, H. J. B. & Line, J. M. The use of Rarefaction Analysis for Estimating Palynological Richness from Quaternary Pollen-Analytical Data. *The Holocene* **2**, 1–10 (1992).
3. Giesecke, T., Ammann, B. & Brande, A. Palynological richness and evenness: Insights from the taxa accumulation curve. *Veg. Hist. Archaeobotany* **23**, 217–228 (2014).
4. Blaus, A. *et al.* Modern Pollen–Plant Diversity Relationships Inform Palaeoecological Reconstructions of Functional and Phylogenetic Diversity in Calcareous Fens. *Front. Ecol. Evol.* **8**, (2020).
5. Birks, H. J. B. *et al.* Does pollen-assemblage richness reflect floristic richness? A review of recent developments and future challenges. *Rev. Palaeobot. Palynol.* **228**, 1–25 (2016).
6. Meltsov, V., Poska, A., Reitalu, T., Sammul, M. & Kull, T. The role of landscape structure in determining palynological and floristic richness. *Veg. Hist. Archaeobotany* **22**, 39–49 (2013).
7. Berglund, B. E., Persson, T. & Björkman, L. Late Quaternary landscape and vegetation diversity in a North European perspective. *Quat. Int.* **184**, 187–194 (2008).
8. Woodbridge, J. *et al.* What drives biodiversity patterns? Using long-term multidisciplinary data to discern centennial-scale change. *J. Ecol.* **109**, 1396–1410 (2021).
9. Feurdean, A., Willis, K. J., Parr, C. L., Tanţău, I. & Fărcaş, S. Post-glacial patterns in vegetation dynamics in Romania: homogenization or differentiation? *J. Biogeogr.* **37**, 2197–2208 (2010).
10. Giesecke, T. *et al.* Postglacial change of the floristic diversity gradient in Europe. *Nat. Commun.* **10**, 5422 (2019).
11. Pielou, E. C. The measurement of diversity in different types of biological collections. *J. Theor. Biol.* **13**, 131–144 (1966).

12. Shannon, C. E. A mathematical theory of communication. *Bell Syst. Tech. J.* **27**, 379–423 (1948).
13. Odgaard, B. V. Palaeoecological perspectives on pattern and process in plant diversity and distribution adjustments: a comment on recent developments*. *Divers. Distrib.* **7**, 197–201 (2001).
14. Odgaard, B. V. Fossil pollen as a record of past biodiversity. *J. Biogeogr.* **26**, 7–17 (1999).
15. van der Knaap, W. O. Estimating pollen diversity from pollen accumulation rates: a method to assess taxonomic richness in the landscape. *The Holocene* **19**, 159–163 (2009).
16. Baselga, A. & Orme, C. D. L. betapart: an R package for the study of beta diversity. *Methods Ecol. Evol.* **3**, 808–812 (2012).
